# Severe Bandemia Is Not Associated With Increased Risk for Adverse Events in General Pediatric Emergency Department Patients

**DOI:** 10.7759/cureus.13303

**Published:** 2021-02-12

**Authors:** Daniel Najafali, Noorvir Kaur, Ikram Afridi, Norhan Abdalla, Leenah Afridi, Iana Sahadzic, Julianna Solomon, Isha Yardi, Quincy K Tran

**Affiliations:** 1 Research Associate Program in Emergency Medicine and Critical Care, University of Maryland School of Medicine, Baltimore, USA

**Keywords:** adverse outcome, bacterial illness, bandemia, general pediatric emergency department patients, severe bandemia

## Abstract

Introduction: The presence of band cells > 10% of the total white blood cell (WBC) count (“bandemia”) is often used as an indicator of serious bacterial illness (SBI). Results from studies of bandemia as a predictor of SBI were conflicting and little is known about the relationship between severe bandemia (SB) and clinical outcomes from SBI in children. We hypothesized that SB (band level > 20%) is not associated with adverse outcomes in an emergency department (ED) pediatric population.

Methods: Medical records from children between the ages of two months and 18 years with SB who presented to a tertiary referral regional hospital were studied. Outcomes were categorized as severe adverse events (SAEs) or moderate adverse events (MAEs). Multivariate logistic regressions were used to assess the association between SB and outcomes.

Results: We analyzed 102 patients. Mean age (standard deviation, SD) was 5.25 (0.5) years, 18 (18%) had MAE, 21 (21%) had SAE, and no patients died. Mean band levels were similar between groups: no adverse events 28 (10) vs. SAE 31 (9) vs. MAE 27 (8), p=0.64. Multivariate logistic regressions showed SB was not associated with any adverse events (odds ratio (OR) 1.04, 95% confidence interval (CI) 0.9-1.1, p=0.27). Non-normal X-ray (XR) (OR 17, 95% CI 3.3-90, p<0.001) was associated with MAE, while non-normal computerized tomography (CT) scan (OR 15.4, 95% CI 2.2-100+, p=0.002) was associated with SAE.

Conclusion: SB was not associated with higher odds of adverse events among the general ED pediatric population. Clinicians should base their clinical judgment on the overall context of history, physical examinations, and other laboratory and imaging data.

## Introduction

Among the pediatric population in the United States, 50,000 cases of severe sepsis occur annually, a number that has been on the rise for a condition that carries a mortality rate of approximately 10% [1]. In 1992, the Society of Critical Care Medicine designated a band cell concentration of more than 10% of the total peripheral white blood cell (WBC) count as one of the criteria for systemic inflammatory response syndrome [2]. Since then, many clinicians have considered an elevated band cell level as a surrogate for serious bacterial illness (SBI).

Bandemia was associated with bloodstream infection in the adult patient population [3]. In the pediatric emergency medicine literature, sustained controversy questions whether a band cell level greater than 10% of the WBC count truly indicates SBI. Jaskiewicz et al., writing in 1994, and Schnadower et al., publishing in 2010, claimed that pediatric patients with bandemia greater than 10% were at risk of SBI [4,5]. In contrast, other author groups in the past reported no association between bandemia greater than 10% and SBI [6-8]. Kuppermann et al. suggested that bandemia at 13% did not differentiate between pediatric patients with culture-proven or laboratory-proven bacterial infections and those with viral infections [8].

Most published studies considered a band level of 10% as the threshold for bacterial infection. There is scant evidence in the pediatric emergency medicine literature about the association between severe bandemia (SB) (>20% band cells) and clinical outcomes. We hypothesized that SB is not associated with an increased risk of adverse events and outcomes. This article was previously posted to the medRxiv preprint server on January 4, 2021.

## Materials and methods

Study design

We conducted a retrospective cohort study using medical records from a single pediatric emergency department (ED) at an urban, regional tertiary referral level one pediatric trauma center. The study was approved by the hospital’s institutional review board.

Patient population and data collection

We created a convenience cohort by first identifying patients who were two months to 18 years of age when they were brought to the ED for evaluation between January and July 2008. All patients during the study period within the aforementioned age range were included. We selected this date because our regional pediatric hospital ceased to perform differential cell count on all patients’ blood samples. Starting on August 1, 2008, differential cell count, and band levels, were only performed by orders of physicians who suspected infection in their patients. As a result, blood samples with bands after August 1, 2008 were most likely performed on patients with high suspicion for infection.

SB was defined as band cell levels ≥ 20 (constituting 20% of the total peripheral WBC count) [9]. Any imaging study (computerized tomography, CT, scan or X-ray (XR) films) interpreted by attending radiologists as suggesting an infectious process would be considered “non-normal” imaging studies.

An investigator who was not blind to the study hypothesis abstracted the data into a standardized form using an Excel spreadsheet (Microsoft Corporation, Redmond, WA). Data were subsequently prepared by other investigators who were blind to the study hypothesis prior to analysis.

Outcomes

The primary outcome in this study was a severe adverse event (SAE) associated with bacterial illness. SAEs were defined as in-hospital death, hospital admission from the index ED visit or within seven days, returning to the ED within seven days, bacteremia (blood culture showing > 10,000 colony-forming units per milliliter), or positive cerebrospinal fluid (CSF, WBC count > 10 cells per high-power field, positive gram stain).

The secondary outcome was any moderate adverse event (MAE) associated with bacterial illness. These events were defined as positive urinalysis (urinary WBC count > 100,000 cells/high-power field, positive nitrite or leukocyte esterase) and bacteria-positive stool cultures. The other outcome was any adverse event (AAE), which is a combination of SAEs and MAEs.

Data analysis

We first performed descriptive analyses to categorize patients in each subgroup. Continuous variables were assessed for normal distribution by the Ryan-Joiner test. Normally-distributed-data were expressed as mean (standard deviation, SD) and compared via the Student’s t-test. Chi-square tests were used to compare groups of categorical data, while analysis of variance (ANOVA) with Holm-Sidak post-hoc tests, were used to compare means between groups of normally-distributed-continuous-data.

We also performed multivariable logistic regressions to assess the association between clinical factors and outcomes (SAE, MAE, or AAE). Statistical analyses were performed with Sigma Plot version 13.0 (Systat Software Inc., San Jose, CA). Two-tailed p-values < 0.05 were considered statistically significant.

## Results

The records of 113 patients treated during the six-month study period indicated SB. We excluded 11 patients from that original group: four because the measured band level was missing from the record, and seven because they were over 19 years of age; therefore, 102 patients were included in the final analysis. Mean (SD) was 5.25 (0.5) years.

Twenty-one patients (21%) had SAEs, 18 (18%) had MAEs, 39 (38%) AAE (Table 1), and 63 (62%) patients did not have AAE. 

**Table 1:** Characteristics of pediatric patients with severe bandemia. Bands were counted as percentage of total WBC count. Abbreviations: CSF, cerebrospinal fluid; CT, computerized tomography scan; ED, emergency department; mL, milliliters; SD, standard deviation; WBC, white blood cell; XR, X-ray radiograph

Characteristics	Results
Total patients, N (%)	102 (100)
Age (years), mean (SD)	5.25 (0.5)
Age groups, N (%)	
Two months – two years	43 (42)
Three years – 18 years	59 (58)
Gender, N (%)	
Male	50 (49)
Female	52 (51)
Any non-normal physical findings, N (%)	47 (46)
Fever, N (%)	38 (37)
WBC (cell/mL), mean (SD)	12 (0.6)
Bands, mean (SD)	29 (0.9)
Non-infectious diagnoses, N (%)	17 (17)
Positive urinalysis result, N (%)	7 (7)
Positive cultures, N (%)	
Blood culture	1 (0.9)
CSF	0 (0)
Stool culture	3 (2)
Urine culture	4 (4)
Imaging studies, N (%)	
Non-normal XR	12 (12)
Non-normal CT	8 (8)
Adverse events, N (%)	
Any adverse event	39 (38)
Moderate adverse event	18 (18)
Severe adverse event	21 (21)
Outcomes, N (%)	
Admission from ED	17 (17)
Return to ED within seven days	4 (4)
Death	0 (0)

**Figure 1:**
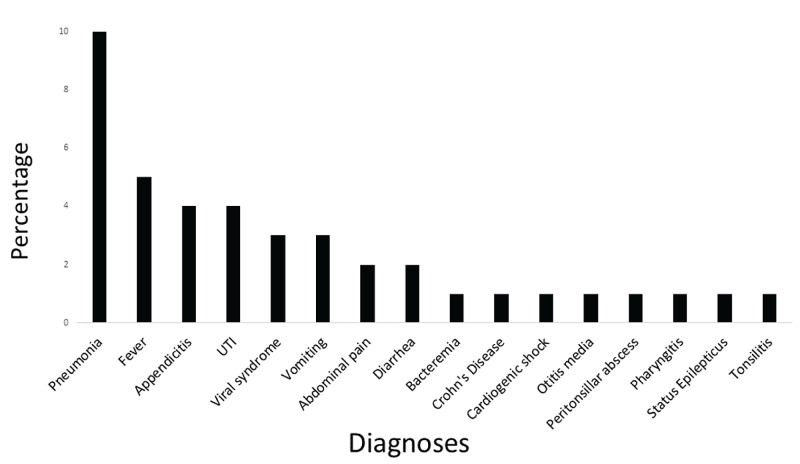
Hospital diagnoses of patients who had any adverse events. Abbreviations: UTI, urinary tract infection

Among the 21 SAE patients, 17 were admitted during the index ED visit and four were discharged during the initial visit but returned to the ED within seven days. There was no mortality and there was no readmission within seven days after the index ED visit. The most common diagnosis for 39 patients with AAE was pneumonia (10%), fever (5%), and appendicitis (4%) (Figure 1).

Characteristics of patients between groups were similar (Table 2). Mean band level for patients with no adverse events was 28 (10). This level was non-statistically similar (p-value=0.64) to patients with SAE, MAE, or AAE with mean band levels of 31 (9), 27 (8), and 29 (9), respectively. Patients with no adverse events had significantly less non-normal XR and CT findings (p<0.001), compared to those with adverse events.

**Table 2:** Characteristics of patients having adverse events. *Bands were defined as percentage of total WBC count. Abbreviations: AAE, any adverse event; CT, computerized tomography scan; mL, milliliters; MAE, moderate adverse event; PE, physical examination; SAE, severe adverse event; SD, standard deviation; WBC, white blood cell; XR, X-ray radiograph

Variables	No Adverse Events (N=63)	SAE (N=21)	MAE (N=18)	AAE (N=39)	P
Age (years), mean (SD)	5 (5)	7 (5)	4 (4)	6 (5)	0.14
Gender, N (%)					0.35
Female	31 (49)	14 (66)	7 (39)	21 (54)	
Male	32 (50)	7 (33)	11 (61)	18 (46)	
Non-normal PE findings, N (%)	29 (46)	13 (62)	5 (28)	18 (4)	0.21
Fever, N (%)	24 (48)	5 (24)	9 (50)	14 (36)	0.40
WBC (10^3^ cell/mL), mean (SD)	11 (5)	13 (8)	14 (6)	14 (7)	0.07
Bands*, mean (SD)	28 (10)	31 (9)	27 (8)	29 (9)	0.64
Non-normal XR finding, N (%)	1 (2)	3 (14)	8 (44)	11 (28)	<0.001
Non-normal CT finding, N (%)	1 (2)	6 (28)	1 (5)	7 (18)	0.002

Multivariate logistic regressions, after adjusted for age, gender, physical examinations, the presence of fever, WBC, bands, XR and CT results, showed that band levels were not associated with SAE (odds ratio (OR) 1.04, 95% CI 0.9-1.1, p-value=0.27), MAE (OR 0.97, 95% CI 0.9-1.1, p-value=0.43), or AAE (OR 1.01, 95% CI 0.9-1.1, p-value=0.49) (Table 3). Non-normal XR was significantly associated with MAE (OR 17, 95% CI 3.3-90, p-value<0.001) and AAE (OR 32, 95% CI 3.5-100+, p-value<0.001). Non-normal CT was associated with SAE (OR 15, 95% CI 2.2-100+, p-value=0.002) and AAE (OR 27, 95% CI 2.5-100+, p-value=0.001).

**Table 3:** Results from multivariate logistic regressions. Abbreviations: AAE, any adverse event; CI, confidence interval; CT, computerized tomography scan; MAE, moderate adverse event; OR, odds ratio; PE, physical examination; SAE, severe adverse event; WBC, white blood cell; XR, X-ray radiograph

Variables	AAE			MAE			SAE		
	OR	95% CI	P	OR	95% CI	P	OR	95% CI	P
Age (years)	0.97	0.8-1.1	0.62	0.97	0.8-1.1	0.58	1.03	0.9-1.2	0.61
Gender	0.91	0.3-2.6	0.85	0.91	0.2-2.5	0.42	0.47	0.1-1.5	0.20
Non-normal PE	0.84	0.3-2.4	0.76	0.84	0.3-2.4	0.16	1.15	0.4-3.7	0.82
Fever	0.68	0.2-2.2	0.52	0.68	0.2-2.2	0.92	0.84	0.2-3.1	0.80
WBC	1.04	0.9-1.2	0.38	1.01	0.9-1.1	0.82	1.03	0.9-1.1	0.45
Bands	1.01	0.9-1.1	0.49	0.97	0.9-1.1	0.43	1.04	0.9-1.1	0.27
Non-normal XR	32	3.5-100+	<0.001	17	3.3-90	<0.001	1.08	0.2-1.2	0.94
Non-normal CT	27	2.5-100+	0.001	1.2	0.1-14	0.91	15.4	2.2-100+	0.002

## Discussion

In our study, based on a general pediatric ED population, SB (band cells constituting more than 20% of the total peripheral WBC count) was not associated with a higher risk of adverse outcomes caused by bacterial illness. None of the patients in our study group died during hospitalization or required hospital readmission within seven days after their initial ED evaluation. Our observations suggested that SB should not be used as a single factor that influences clinical decisions.

Further studies in pediatric patients are needed to confirm our finding that SB was not associated with clinical outcomes. However, our finding was consistent with previous studies involving adult ED patients. Ward et al. [10] reported that median bandemia levels for septic adult survivors were 9%, compared to 17% for septic non-survivors (p-value=0.766) and increasing band level was not associated with increased odds of death (95% CI 0.99-1.028, p-value=0.354).

SB was noted in patients with other inflammatory causes for admission, such as status epilepticus and cardiogenic shock from congenital heart disease, suggesting that bandemia serves as a good inflammatory marker but might not be a good marker for severe infection or adverse clinical outcomes. In contrast, in a study of children with appendicitis [11], Whyte and associates reported that nonoperative management failed more often in those with bandemia greater than 11% than in those with bandemia less than 10%, which resulted in longer hospital length of stay (LOS) (17 vs. 9 days). The difference between our findings and Whyte et al.’s could be explained by the low prevalence of appendicitis (4%) in our study population or a low prevalence of SBI among our ED pediatric population with SB.

Four (4%) patients in the SAE group returned to the ED within seven days after their initial ED visit because of abdominal pain, vomiting, or fever. None were re-admitted to the hospital. This rate of ED return is similar to those documented in previous reports [12,13]. Most pediatric patients return to EDs because their disease progresses, and up to 30% of them are admitted [14]. In our population, returning patients with SAE were not admitted at the second visits, possibly because the sample of returning patients was small (4%) and because of the poor association between SB and these patients’ diseases.

Our study also found no association between SB and a higher risk of MAE caused by bacterial illness. The most common diagnoses for patients in the MAE group were pneumonia (8%) and urinary tract infection (UTI) (4%). Patients in this group were discharged with antibiotics and did not return to the ED or get re-admitted to the hospital within the subsequent seven days. Thus, without other indicators such as hypoxia, SB did not affect outcomes among pediatric patients with these infections.

Limitations

Our study has several limitations. In addition to those inherent to retrospective studies, we did not have an age-matched control group with band counts constituting <20% of the total peripheral WBC count and thus could not compare the rates of adverse events between the study group and a control group. Therefore, we were not able to calculate sensitivity or specificity of SB as predictors of clinical outcomes. Furthermore, as a single-center study, we might have missed patients who returned to EDs at other hospitals, although this missing rate might be low because the study hospital is the only tertiary referral pediatric hospital in the region.

This study provides information that will be helpful in the design of future investigations of bandemia and outcomes among pediatric patients. Future studies with a higher percentage of surgical patients will provide more conclusive information regarding SB in medical vs. surgical patients, such as those with appendicitis, which might be associated with higher levels of bandemia and different outcomes from those of medical pediatric patients.

## Conclusions

SB is an inflammatory marker and is not associated with higher risks of adverse events caused by bacterial illness among ED pediatric patients. SB should not be used as a single factor in clinical decisions. Clinicians should base their clinical judgment on the overall context of physical examination findings, other laboratory data, and imaging studies.
